# Lack of ZNF365 Drives Senescence and Exacerbates Experimental Lung Fibrosis

**DOI:** 10.3390/cells11182848

**Published:** 2022-09-13

**Authors:** Juan Urista, Mariel Maldonado, Fernanda Toscano-Marquez, Remedios Ramírez, Yalbi Itzel Balderas-Martínez, Carina Becerril, Yair Romero, Moisés Selman, Annie Pardo

**Affiliations:** 1 Facultad de Ciencias, Universidad Nacional Autónoma de México, Ciudad de Mexico 04510, Mexico; 2 Instituto Nacional de Enfermedades Respiratorias, “Ismael Cosío Villegas”, Ciudad de Mexico 14080, Mexico

**Keywords:** zinc finger protein ZNF365, pulmonary fibrosis, TGFβ-1, cellular senescence

## Abstract

Idiopathic pulmonary fibrosis (IPF) is characterized by aberrant activation of the alveolar epithelium, the expansion of the fibroblast population, and the accumulation of extracellular matrix. Global gene expression of human lung fibroblasts stimulated with TGFβ-1, a strong fibrotic mediator revealed the overexpression of ZNF365, a zinc finger protein implicated in cell cycle control and telomere stabilization. We evaluated the expression and localization of ZNF365 in IPF lungs and in the fibrotic response induced by bleomycin in WT and deficient mice of the orthologous gene *Zfp365*. In IPF, ZNF365 was overexpressed and localized in fibroblasts/myofibroblasts and alveolar epithelium. Bleomycin-induced lung fibrosis showed an upregulation of Zfp365 localized in lung epithelium and stromal cell populations. *Zfp365* KO mice developed a significantly higher fibrotic response compared with WT mice by morphology and hydroxyproline content. Silencing *ZNF365* in human lung fibroblasts and alveolar epithelial cells induced a significant reduction of growth rate and increased senescence markers, including Senescence Associated β Galactosidase activity, p53, p21, and the histone variant γH2AX. Our findings demonstrate that ZNF365 is upregulated in IPF and experimental lung fibrosis and suggest a protective role since its absence increases experimental lung fibrosis mechanistically associated with the induction of cell senescence.

## 1. Introduction

Idiopathic pulmonary fibrosis (IPF) is a chronic, progressive, irreversible, and lethal lung disease of unknown etiology that is associated with high morbidity and mortality. Although great efforts have been made in recent years trying to elucidate the molecular and cellular mechanisms underlying its pathogenesis and progression, there are still many questions left unanswered. IPF is an epithelial-fibroblastic disease, where the aberrant activation of alveolar epithelial cells results in the secretion of multiple profibrotic mediators such as TGFβ-1, TNFα, and PDGF, among others. These factors induce the migration and proliferation of fibroblasts and their differentiation to myofibroblasts [1,2,3].

TGFβ−1 is one of the most important mediators that orchestrate fibrotic lung disease. Global gene expression analysis revealed that ZNF365, a zinc finger protein, is overexpressed by the effect of TGFβ-1 in lung fibroblasts derived from control subjects and IPF patients [4,5]. However, its role in this disease has not been explored.

Studies reported in the literature regarding the role of ZNF365 are scant; it has been observed to be associated with DNA double-strand break repair mechanisms through physically interacting with the exonuclease MRE11 and Poly ADP Ribose Polymerase 1 (PARP1) and regulating stalled replication forks which result from DNA double-strand breaks (DSBs) [6]. It has also been described as a target of p53 in cells undergoing telomeric stress, avoiding the formation of anaphase DNA bridges [7]. In murine models lacking the orthologue of *ZNF365*, known as Zfp365, although no evident phenotype has been described, there are some defects in neuronal migration from the brain’s subventricular zone to the cerebral cortex [8,9]. Another important function described for this molecule is its involvement in the correct chromosome segregation during mitosis by interacting with centrosome proteins such as Nde1 [10].

Many zinc finger proteins have been described earlier participating in IPF, for example, Gli transcription factors from the Sonic Hedgehog (SHH) signaling pathway. Other authors have described the zinc finger protein family members as differentially methylated in IPF [11,12].

Thus, this study aimed to evaluate the expression and cellular localization of ZNF365 in IPF lungs and to analyze the role of ZNF365 in the lung fibrotic response induced by bleomycin in WT mice and mice deficient in the orthologous gene. We also examined the effect of ZNF365 silencing on normal human lung fibroblasts and alveolar epithelial cells in vitro and analyzed its putative mechanism.

## 2. Results

### 2.1. ZNF365 Is Increased in IPF Lung Tissue, and It Is Located in Fibroblastic Foci and Alveolar Epithelial Cells

**Figure 1:**
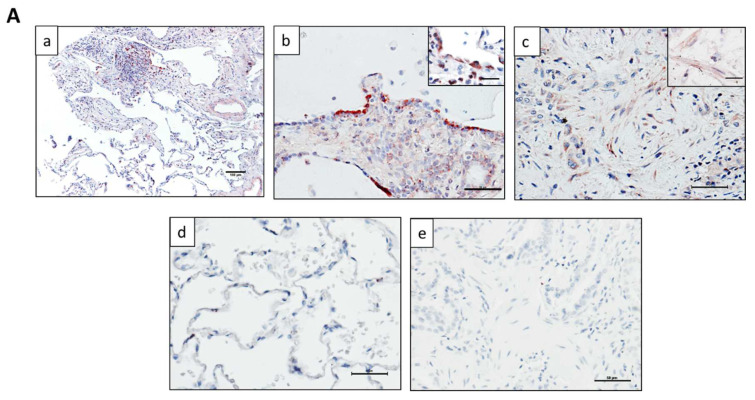

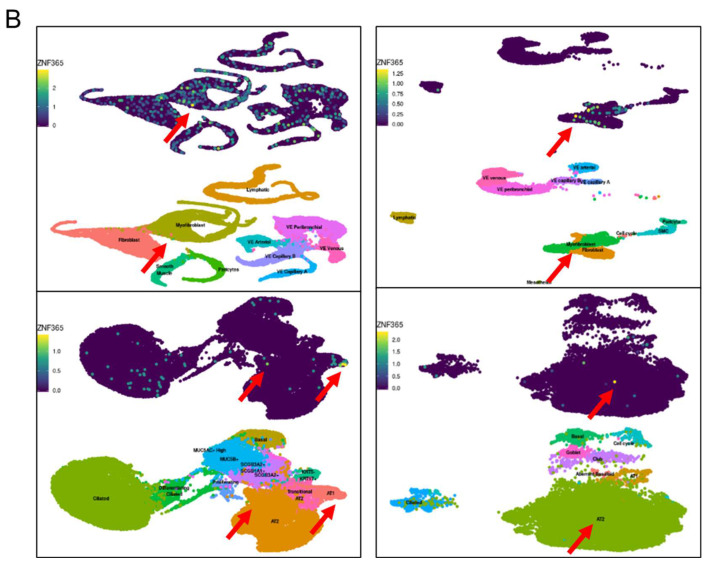
ZNF365 is expressed in IPF lung tissues. (**A**) ZNF365 is overexpressed in IPF lung tissues (*n* = 5), and human control lungs (*n* = 2) were stained for immunohistochemistry with the specific antibody. (**a**) Immunoreactive protein was located in fibroblast foci and alveolar epithelial cells (scale bar 100 μm). (**b**,**c**) Representative epithelial cells and fibroblasts in two different magnifications (scale bars 50 and inset 25 μm). (**d**) Immunostaining of lung tissue from control donor (scale bar 50 μm). (**e**) Immunostaining of IPF lung tissue without the primary antibody (scale bar 50 μm). (**B**) ZNF365 expression according to scRNAseq experiments from IPF Cell Atlas. (http://ipfcellatlas.com/, 9 August 2022).

We first determined the expression and localization of ZNF365 in IPF and control lung tissues by immunohistochemical (IHC) staining. As shown in Figure 1A, panels a, b, and c, immunoreactive ZNF365 was present in alveolar epithelial cells delineating a cystic space (b) and in fibroblasts from fibroblastic foci in IPF lungs (c). In contrast, in normal lung tissue, ZNF365 staining was virtually absent (panel d). No staining was observed when the specific antibody was omitted (panel e). These findings indicate that ZNF365 is overexpressed in IPF lungs and localized predominantly in fibroblasts from fibroblastic foci and alveolar epithelium.

*In silico* analysis of single-cell transcriptional atlas of the lungs of IPF patients corroborated that ZNF365 is mainly expressed in fibroblasts, myofibroblasts, and alveolar epithelial type 1 and type 2 cells, as well as other epithelial and fibroblasts “rare” subpopulations (Figure 1B).

### 2.2. ZNF365 Mouse Orthologue, Zfp365, Is Overexpressed in Lungs from Mice Treated with Bleomycin

**Figure 2:**
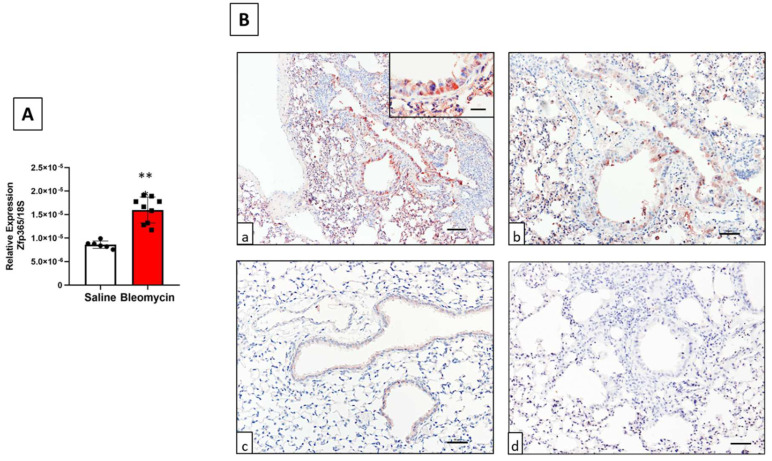
Zfp365 is overexpressed in lungs from mice exposed to bleomycin after 21 days. (**A**) Zfp365 expression by qPCR in lungs of mice treated with bleomycin (*n* = 9) or saline solution (*n* = 6). The graph represents the mean ± SD. ** *p* < 0.001 by a two-tailed Student’s *t*-test with Welch’s correction. (**B**) IHC of lung tissues from mice instilled with saline solution or bleomycin. (**a**,**b**) Bleomycin-treated mice showed immunoreactive protein primarily in alveolar and bronchial epithelial cells and some stromal cell populations (*n* = 2) (scale bars 100 μm, 50 μm, and inset 25 μm). (**c**) Immunostaining in control mice instilled with saline solution (*n* = 2) (scale bar 50 μm). (**d**) Immunostaining of bleomycin-treated mice without the primary antibody (scale bar 100 μm).

We then evaluated the expression of the gene orthologue, Zfp365, by qPCR analysis of lung tissue and the cellular localization by IHC staining in WT mice after bleomycin injury. At the mRNA level, a significantly increased expression of *Zfp365* in lungs derived from bleomycin instilled mice at 21 days was observed (*p* < 0.001) (Figure 2A). This was corroborated by IHC (Figure 2B), where Zfp365 is overexpressed in bleomycin-instilled mice and localized in alveolar and bronchial epithelial cells and some stromal cell populations (panel a). Zfp365 was nearly absent in the lungs of mice instilled with saline solution (panel b). No specific signal was observed when the specific antibody was absent (panel c).

### 2.3. TGFβ-1 Stimulates the Expression of ZNF365 in Normal and IPF Lung Fibroblasts and in the A549 Epithelial Cell Line

**Figure 3:**
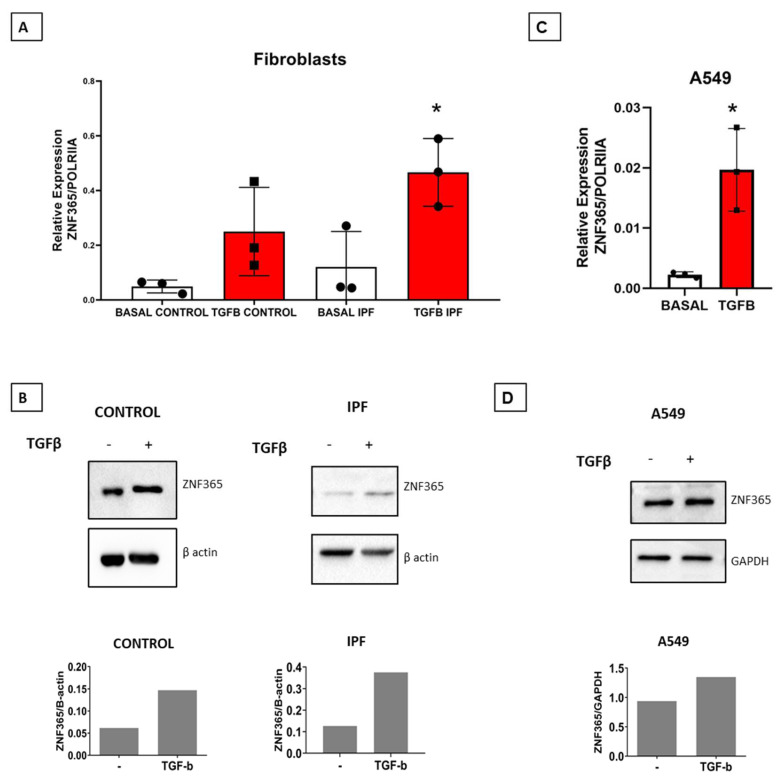
TGFβ-1 induces the overexpression of ZNF365. (**A**) ZNF365 expression in control (*n* = 3) and IPF lung fibroblasts (*n* = 3) by qPCR after 24 h of TGFβ-1 stimulation. (**B**) Western Blot and the corresponding densitometric analysis; β actin was used as the internal loading control. (**C**) ZNF365 expression in A549 cells and after 24 h of TGFβ-1 stimulation (*n* = 3). (**D**) Western blot and corresponding densitometric quantification; GAPDH was used as the internal loading control. * *p* < 0.05 by a two-tailed Student’s *t*-test with Welch’s correction.

To corroborate results obtained with global gene expression [4,5], we exposed fibroblasts derived from normal human and IPF lungs to TGFβ-1. This potent profibrotic mediator plays a relevant role in lung fibrosis. As shown in Figure 3A,B, ZNF365 was significantly overexpressed at mRNA (*p* < 0.05 and protein levels in both normal and IPF fibroblasts after TGFβ−1 stimulation (10 ng/mL) for 24 h. Since ZNF365 was also found overexpressed in alveolar epithelium from IPF lungs, we examined its expression in the A549 epithelial cell line stimulated with TGFβ-1 by qPCR and Western blot. As shown in Figure 3C,D, ZNF365 was significantly upregulated at both mRNA (*p* < 0.05) and protein levels.

### 2.4. Zfp365 KO Mice Display an Exacerbated Fibrotic Response to Bleomycin

**Figure 4:**
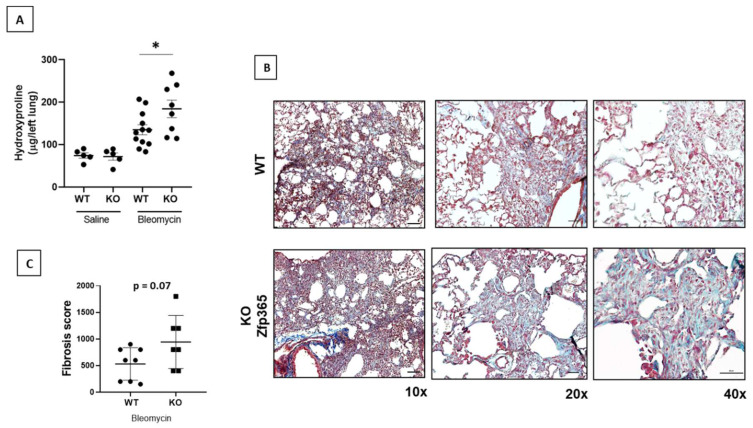
An exacerbated fibrotic response was observed in Zfp365-deficient mice. (**A**) Lung hydroxyproline content at 21 days after bleomycin instillation of Zfp365 KO mice (*n* = 8) and WT (*n* = 12). (**B**) Representative light microscopy images of Masson’s trichrome stained lung tissue sections in three different magnifications (scale bars 100 and 50 μm) from WT and Zfp365 KO mice 21 days after bleomycin instillation. (**C**) Fibrosis score for grading lung histopathological changes. Results are shown as mean ± SEM. Statistical significance was determined by the Student’s *t*-test with Welch’s correction (* *p* < 0.05).

To explore the role of Zfp365 in vivo, WT C57BL/6 and Zfp365 deficient mice (Supplementary Figure S1) were intratracheally instilled with bleomycin or saline solution as controls. After 21 days, mice were sacrificed, and histological changes and hydroxyproline content were assessed. To evaluate the fibrotic response quantitatively, we measured hydroxyproline content in both WT and Zfp365 KO mice lungs. As illustrated in Figure 4A, bleomycin-instilled Zfp365 deficient mice displayed a significant increase in hydroxyproline content compared with WT mice (184.3 + 58.8 versus 135.2 + 40.5 μg/lung, *p* < 0.05). Zfp365 KO mice showed an accumulation of collagen fibers as indicated by Masson’s trichrome staining compared with their WT counterparts (Figure 4B). The semiquantitative fibrosis score showed a tendency to increase in Zfp365 deficient mice, although it did not reach statistical significance, likely because of the heterogeneity of the lesions and the sample size (Figure 4C).

### 2.5. ZNF365 Silencing Induces a Senescent Phenotype in Fibroblasts and Epithelial Cells

**Figure 5:**
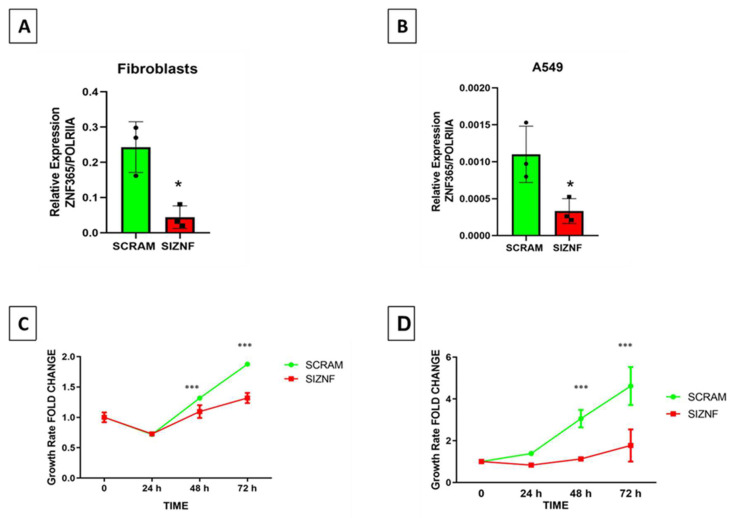
Silencing of ZNF365 reduces fibroblasts and A549 cells growth rate. (**A**) Expression of ZNF365 was examined by qPCR in normal human lung fibroblasts silenced. (**B**) Expression of ZNF365 by qPCR analysis in silenced A549 cells. “SCRAM” corresponds to the scrambled control sequences and “SIZNF” to specific siRNA sequences for ZNF365. (**C**) Growth rate assay of normal human lung fibroblasts using WST-1 reagent. (**D**) Growth rate assay of A549 cells using WST-1 reagent. Graphics represent three independent experiments by triplicate (qPCR) and curves represent one of three independent experiments by quadruplicate (WST-1) (mean ± standard deviation). * *p* < 0.05, by a two-tailed Student’s *t*-test with Welch’s correction; *** *p* < 0.001 by two-way ANOVA with Bonferroni’s posttest.

Considering that *Zfp365* deficient mice developed severe lung fibrosis after bleomycin instillation, we decided to explore in vitro the functional behavior of lung fibroblasts and epithelial cells after silencing this zinc finger protein. Using specific siRNA sequences and their respective scrambled control sequences, we show with qPCR analysis that *ZNF365* mRNA was significantly downregulated at 48 h in both cell types (*p* < 0.05) (Figure 5A,B). After silencing the gene in lung fibroblasts and in A549 epithelial cells, we performed a growth rate analysis using WST-1. A significant decrease was observed in silenced cells for ZNF365 at 48 and 72 h (*p* < 0.001) (Figure 5C,D).

As growth rate involves proliferation and cell death, we measured the early and late apoptosis using 7-AAD and Annexin V staining by flow cytometry. After an apoptotic stimulus of bleomycin (30 mU) for 48 h, no significant differences in A549 cells silenced for ZNF365 and those with scrambled control sequences were found. Also, after an apoptotic stimulus with staurosporine (0.1 and 0.5 μM) for 1 h, no significant differences were observed in human lung fibroblasts silenced for ZNF365 (Supplementary Figure S2). Additionally, we investigated if fibroblast silencing of ZNF365 affects Col1a1 and Col1a3 expression. Our results showed an increase in both gene expressions (Supplementary Figure S3).

**Figure 6:**
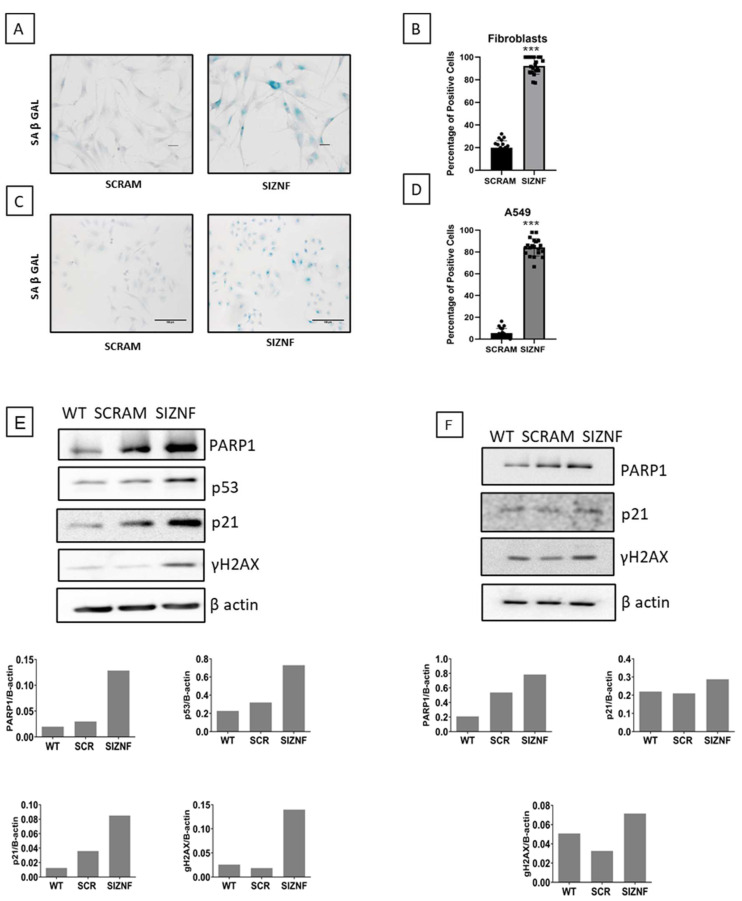
Normal human lung fibroblasts and A549 cells acquire a senescent phenotype after *ZNF365* silencing. Senescence-associated β galactosidase activity (SA-β Gal) staining photomicrograph of normal human lung fibroblasts (**A**) and A549 epithelial cells (**C**) transfected with scrambled control sequences “SCRAM” or with specific siRNA sequences for ZNF365 “siZNF” (Scale bar 100 μm). (**B**,**D**), Percentage of positive SA-β Gal staining. Western blot analysis of senescent markers in ZNF365 silenced normal human lung fibroblasts (**E**) and A549 cells (**F**); β actin was used as the internal loading control. Densitometric analyses are shown. (**B**,**D**) graphics represent the mean ± SD of one experiment with two technical replicates and ten random bright microscopic fields counted for each replicate. *** *p* < 0.001 by a two-tailed Student’s *t*-test with Welch’s correction.

The findings that silencing of ZNF365 reduced cell growth rate without affecting cell death suggested that senescence might be involved as a mechanism contributing to the functional effect of this molecule. Accordingly, we analyzed the impact of silencing ZNF365 on the expression of several senescent markers. Senescence-associated beta-galactosidase assay (SA-βGal) showed increased activity in normal human lung fibroblasts and A549 cells silenced for ZNF365 (Figure 6A,C). As shown in Figure 6B,D, the percentage of SA-βGal positive cells was significantly higher in the silenced cells after counting ten random bright microscopic fields (20× augmentation) by two technical replicates (*p* < 0.001). The analysis by WB of other well-known biomarkers of senescence as p53, p21, γH2AX, and PARP1 [13] exhibited an increased expression of these four proteins in human lung fibroblasts, and of PARP1, p21, and γH2AX in A549 epithelial cells (Figure 6E,F).

In addition, we examined the effect of TGFβ-1 stimulation on the expression of senescence markers in ZNF365-silenced human lung fibroblasts and A549 epithelial cells. As shown in Supplementary Figure S4, TGFβ-1 increased the expression of p21 and p53 in ZNF365-silenced cells.

### 2.6. Reduction of ZNF365 Expression in Normal Human Lung Fibroblasts and A549 Epithelial Cells Is Observed by Doxorubicin Treatment

**Figure 7:**
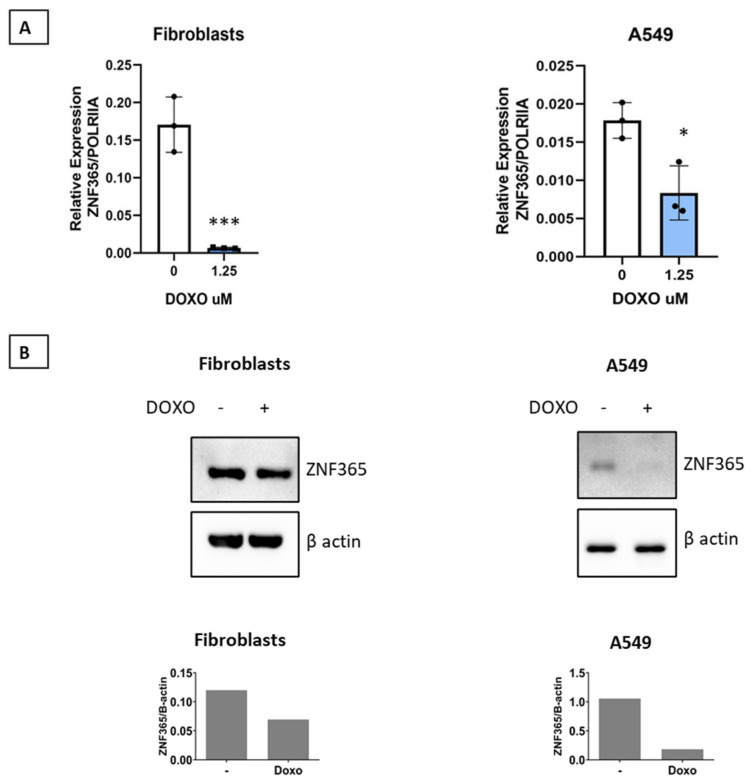
ZNF365 expression is significantly reduced by doxorubicin treatment. (**A**) Doxorubicin treatment reduces ZNF365 expression in normal human lung fibroblasts and A549 cells. (**B**) Western Blot analysis showing ZNF365 downregulation after 48 h of doxorubicin treatment; β actin was used as the internal loading control. Densitometric analysis is shown. Graphics represent the mean ± SD of one of two independent experiments. * *p* < 0.05, and *** *p* < 0.001 by two-tailed Student’s *t*- test.

Doxorubicin provokes DNA damage by inhibiting topoisomerase II and has a senescence-promoting effect in different cell types. We found that stimulation of normal human lung fibroblasts or A549 epithelial cells for 48 h with doxorubicin (1.25 μM) significantly reduces ZNF365 expression both at the mRNA (*p* < 0.001, *p* < 0.05) and protein levels (Figure 7A,B).

## 3. Discussion

IPF is an age-dependent and devastating disease [1,2], with complex and as yet poorly understood disease mechanisms.

TGFβ-1 has long been recognized as one of the critical molecules driving fibrosis in different organs and tissues. A great variety of genes are regulated by TGFβ-1 directly or indirectly (e.g., through the crosstalk with other pathways) [14]. Among all these genes, the zinc finger protein ZNF365 was one of the most upregulated genes in basal and IPF lung fibroblasts after TGFβ-1 stimulation when compared with untreated control fibroblasts [4,5].

*ZNF365* is an essential target whose activation by p53 in cells with critically short telomeres contributes to genomic stability. Moreover, in cells with telomere dysfunction, one of the most strongly upregulated genes upon reconstitution of p53 activity is *ZNF365* [7]. This function suggests that the upregulation of ZNF365 in IPF cells may represent the consequence of exaggerated shortening of telomeres, which has been widely demonstrated in epithelial cells and fibroblasts, usually associated with senescence and exerted by these cells as a defensive mechanism [15,16]. When we explored single-cell expression data downloaded from GEO accession number GSE136831 (see Supplementary Methods), we observed several types of epithelial cells, including basaloid, express this zinc finger protein, and senescent markers (Supplementary Figure S5). However, the putative role of ZNF365 in IPF and other fibrotic lung disorders characterized by short and dysfunctional telomeres [2] has not been examined.

Our study demonstrated that ZNF365 is upregulated in IPF lungs compared to healthy lungs. It was found in some fibroblastic foci and alveolar epithelial cells, which have been primarily characterized as the main cellular effectors in IPF physiopathology. As a proof of concept, we explored the IPF Cell Atlas web page (http://ipfcellatlas.com/ accessed on 9 August 2022) [17,18,19,20] and identified some fibroblasts, myofibroblasts, and epithelial cell populations that overexpress *ZNF365*. We validated these results with qPCR and WB analysis, demonstrating that after 24 h of TGFβ-1 stimulation, ZNF365 is overexpressed in normal human lung and IPF-derived fibroblasts. Given the strong ZNF365 signal in alveolar epithelial cells in IPF lung tissues, we also measured its expression in the A549 epithelial cell line by qPCR and WB, corroborating that ZNF365 is upregulated in these cells by the effect of TGFβ-1.

The expression level of ZNF365 was also examined in lung homogenates from bleomycin-treated mice at day 21 (fibrotic phase), where we found that *Zfp365*, the gene orthologue of *ZNF365*, was also overexpressed compared to the saline controls. Moreover, paralleling the results in IPF, the protein’s location was found mainly in alveolar and bronchial epithelial cells and some stromal populations. In this context, it is well-known that, although useful for many fibrosis-related questions, the bleomycin-induced lung fibrosis model has limitations regarding understanding the progressive nature of human IPF. Among them, the development of fibroblastic foci characteristic of IPF, where we markedly found the expression of ZNF365, is challenging to observe in this experimental model. When we explored the lung fibrotic response in Zfp365-deficient mice after bleomycin installation, we found that these mice displayed an augmented fibrotic response when compared to wild-type animals as assessed by histological analysis and measurement of hydroxyproline content, suggesting that this zinc protein could have an antifibrotic effect.

Although at first sight it seems paradoxical that KO mice develop a higher level of lung fibrosis, while this zinc finger protein is upregulated in IPF, there are many examples of antifibrotic molecules overexpressed in IPF lung tissues that, when downregulated in experimental models, provoke an increased fibrotic response [21,22,23]. This singularity can be explained in the context that during the development of IPF, numerous profibrotic mediators are upregulated, and the increased expression of some antifibrotic molecules likely represents a defensive but insufficient mechanism.

To gain a deeper insight into the possible mechanisms, we explored in vitro the potential function of ZNF365 in human lung fibroblasts and the A549 epithelial cell line, transiently knocking down the gene. We investigated whether the silencing of *ZNF365* in human lung fibroblasts and A549 epithelial cells had any significant effect on cell cycle regulation. Our results showed that ZNF365 silenced cells displayed a substantial reduction of cell growth, with no effect on cell death. Similar results were described with the loss of ZNF365 in the U2OS cell line, resulting in an arrest in the G1/S phase and delayed mitotic progression [6]. This observation suggested that the deficiency of this gene could probably be related to the induction of a senescent phenotype. For this purpose, we examined SA-β Gal activity and measured the expression of proteins considered general biomarkers of cellular senescence in human lung fibroblasts and A549 epithelial cells [24,25]. We found a significantly increased percentage of SA-β Gal positive cells in both cell types silenced for ZNF365. Likewise, an increased expression of PARP1, p21, and γH2AX was observed by Western blot in both cell lines. Importantly, cellular senescence in human lung fibroblasts and alveolar epithelial cells has been recently associated as one of the major drivers in the pathogenesis of IPF [14,26,27]. Cellular senescence is characterized by the inability to proliferate and the production of an abundant pro-fibrotic and pro-inflammatory secretome.

Doxorubicin has long been recognized as a DNA damaging agent as it inhibits topoisomerase II activity, and its role as a senescence-promoting drug has also been described [13,28,29]. We evaluated whether doxorubicin affected the expression of ZNF365 in human lung fibroblasts and A549 epithelial cells, and found a significant decrease in ZNF365 expression in both cell types. Interestingly, doxorubicin also causes significant degradation of PARP1, one of the top downregulated genes when cells are exposed to different senescent promoting stimuli, such as replicative exhaustion, ionizing radiation, doxorubicin treatment, or HRAS^G12V^ overexpression [13]. Interestingly, doxorubicin treatment in lung cancer cells promoted the upregulation of miR-7-5p, which targets PARP1. This same microRNA is predicted from the miRTarBase to target ZNF365. We can speculate that the observed reduction in ZNF365 expression following doxorubicin treatment in our study could result from the same miRNA (senescence-associated miRNA [30]) or, since both molecules physically interact, they could work through feedback loops.

Silencing ZNF365 alone provoked cellular senescence in human lung fibroblasts and A549 cells. It also promoted PARP1 upregulation, which could be a compensation mechanism as ZNF365 is an important molecule for DNA repair [6].

In summary, we have discovered that ZNF365 is upregulated in hyperplastic alveolar epithelial cells and fibroblast foci in IPF lung, as well as in a mice experimental pulmonary fibrosis model, likely as a defense mechanism. The putative antifibrotic protective role of ZNF365 seems to be associated with the regulation of cellular senescence. Indeed, further research is necessary to understand better its functions, which will improve our knowledge about the pathogenesis of this disease.

## 4. Material and Methods

### 4.1. Human Samples

The research protocol was approved by the Ethics Committee of Instituto Nacional de Enfermedades Respiratorias Ismael Cosio Villegas in Mexico City (Identification code: (#B15-18)). Formalin-fixed, paraffin-embedded tissue samples from surgical lung biopsies were used for immunohistochemical analysis. Lung samples from five patients with IPF and two controls were used. IPF diagnosis was undertaken by the Interstitial Lung Disease Program of the INER according to the ATS/ERS/ALAT guidelines.

### 4.2. Immunohistochemistry

Immunohistochemical analysis was performed, as we describe next. Briefly, lung tissues derived from control subjects and IPF patients were deparaffinized, rehydrated, and incubated for 30 min with hydrogen peroxide diluted in methanol to eliminate endogenous peroxidase activity. Antigen retrieval was performed using citrate buffer Ph6. Tissues were blocked using Universal Blocking Solution (Biogenex, San Ramon, CA, USA) for 10 min and then incubated with fetal bovine serum (Gibco, Waltham, MA, USA) for 30 min. Samples were incubated at 4 °C overnight with the ZNF365 primary antibody (Thermo Scientific, PA5-40514 (1:50), Waltham, MA, USA). After primary antibody incubation, lung tissues were incubated with biotinylated secondary antibody (Biogenex, San Ramón, CA, USA) for 20 min. Samples were incubated with streptavidin coupled with horseradish peroxidase-conjugated secondary antibody (Biogenex, San Ramon, CA, USA) for 20 min. Immunodetection was performed with acetate buffer containing 0.05% H_2_O_2_ and 3-Amino-9-ethyl carbazole (AEC) (Biogenex, San Ramon, CA, USA) chromogen. The sections were counterstained with hematoxylin. The primary antibody was replaced by nonimmune serum for negative control slides. Images were taken with a Nikon microscope and were analyzed with NIS-Elements Software.

### 4.3. Cell Culture

Normal human lung fibroblast cell lines NHLF (Lonza Clonetics, New York, NY, USA), LL24 from ATCC (CCL151), and HPF (Promocell, Waltham, MA, USA) were used. Primary cultures of lung fibroblasts from control donors and IPF patients were obtained as previously described [4] and were used between passages 4 to 11. HPF lung fibroblasts were used for functional and senescence experiments. A549 cells were purchased from ATCC (CCL-185). Cells were cultured with HAM’s F12 medium (Gibco, Waltham, MA, USA) supplemented with 10% FBS and 1% antibiotic/antimycotic (Sigma Aldrich, Saint Louis, MO, USA). Cells were incubated at CO_2_–95% air at 37 °C as described elsewhere [4].

### 4.4. TGFβ-1 and Doxorubicin Stimulation

At 70% confluence, human lung fibroblasts were serum-starved for 24 h and A549 epithelial cells for 48 h and then stimulated with TGFβ-1 (R & D, Minneapolis, MN, USA) at a final concentration of 10 ng/mL in Ham’s F12 with low serum (0.1%). For doxorubicin (Sigma, Saint Louis, MO, USA) stimulation, cells were seeded at 50% confluence. After 24 h, the medium was removed and replaced with Ham’s F12 medium with a low serum concentration (2% for A549 and 1% for normal human lung fibroblasts) and [1.25 μM] doxorubicin was added.

### 4.5. Real-Time Polymerase Chain Reaction (PCR)

RNA was extracted using a Trizol reagent (Invitrogen, Carlsbad, CA, USA). One μg RNA was used for RT-PCR using Verso cDNA Synthesis Kit (Thermo Scientific, Waltham, MA, USA). Quantitative PCR (qPCR) was performed using specific Taqman probes (Applied Biosystems, Waltham, MA, USA) for *ZNF365* (Hs00921620_m1), *Zfp365* (Mm00618458_m1), *PARP1* (Hs00242302_m1); *18S* (Hs03003631_g1) and *POLR2A* (Hs00172187_m1). Lightcycler 480 (Roche, Basel, Switzerland) thermal cycler was used for qPCR experiments, and 2^−∆Ct^ was used for data analysis.

### 4.6. Western Blot

Total lysates from lung fibroblasts and A549 cells obtained with RIPA lysis buffer (Saint Louis, MO, USA) were loaded under denaturing conditions onto 10% to 12% SDS/PAGE polyacrylamide gels. Proteins were transferred into nitrocellulose membranes (BioRad, Hercules, CA, USA) and then were blocked using 5% skim milk diluted in 0.05% TBS-T for 1 h. The following primary antibodies were used overnight: for ZNF365 (Abcam, ab157459 (1:500) and ab22890 (1:200), Cambridge, MA, USA), PARP1 (Cell Signaling, (1:500), Danvers, MA, USA), p21 (Santa Cruz, sc-6246, (1:500) Santa Cruz, CA, USA), γH2AX (Abcam, ab81299 phosphorylation of S139, (1:500), Cambridge, MA, USA) p53 (R&D, AF1355, (1:2000), Minneapolis, MN, USA), as well as β actin (Sigma Aldrich, AC-74 (1:10,000), Saint Louis, MO, USA) and GAPDH (Thermo Scientific, PA1-987 (1:2000), Waltham, MA, USA). After TBS-T washing, membranes were incubated with specific HRP-associated secondary antibodies. They were developed with Luminol reagent SuperSignal West Femto Maximum Sensitivity Substrate (Thermo Scientific) using ChemiDoc Gel Documentation System (BioRad, Hercules, CA, USA). Images were analyzed using ImageLab software (BioRad, Hercules, CA, USA), and densitometric analyses were performed.

### 4.7. ZNF365 Silencing

Specific siRNA and Scrambled Negative Control sequences were used in silencing ZNF365 (Origene #SR307854). Briefly, normal human lung fibroblasts and A549 cells were plated on 6, 12, 24, and 96 well plates at a maximum confluence of 70%. Specific siRNA and Scrambled Control sequences were mixed with cells using lipofectamine RNAiMAX (Invitrogen) before plating at a final concentration of 20 nM for both. Cells were used for RNA, protein, growth rate, and SA-β Gal experiments.

### 4.8. Growth Rate Experiments

A549 cells (2 × 10^3^ cells/per well) and normal human lung fibroblasts (5 × 10^3^ cells/per well) were plated on 96 well plates with the mixture of specific siRNAs and Scrambled Negative Control sequences in Ham’s F12 medium and HPF specific medium. After 24 h, 48 h, 72 h, and 96 h, cells were incubated with WST-1 reagent (Roche) and was used according to the manufacturer’s recommendations. Absorbance was measured in Synergy (BioTek, Winooski, VT, USA) equipment at 450 nm and the reference at 620 nm.

### 4.9. Apoptosis

A549 cells previously silenced for ZNF365 (24 h before) were seeded at 70% confluency and stimulated with 30 mU/mL of bleomycin (Cayman) in serum-free medium for 48 h. Normal human lung fibroblasts were seeded at 70% confluency, previously silenced (48 h before) for ZNF365, and stimulated with [0.1 and 0.5 μM] for 1 h. Cells were harvested and stained with PE Annexin V (556421, BD Biosciences, San Jose, CA, USA) and 7-aminoactinomycin D (7AAD, 559925, Biolegend, San Diego, CA, USA). Cells were analyzed by flow cytometry in a FACSAria flow cytometer (BD Biosciences). Results were analyzed with FlowJo 7.8 software (FlowJo, Ashland, OR, USA, Becton Dickinson, and Company).

### 4.10. Senescence Associated-β Galactosidase Activity Assay

Cells were set on coverslips, fixed, and stained with the galactosidase activity kit according to the manufacturer’s instructions (9860S, Cell Signaling). Nuclei were counterstained with hematoxylin. Photographs were taken using an inverted microscope (Nikon, Tokyo, Japan) and were analyzed with NIS-Elements Software. Finally, positive cells were determined by counting β galactosidase positive cells over total cells per field from two technical replicates and ten random bright microscopic fields counted for each replicate. 

### 4.11. Murine Model of Pulmonary Fibrosis

Animal use complied with the relevant guidelines and regulations for animal care, and the protocol was approved by the Bioethics Committee of INER (Identified Code: B-1518: Date October 2018). Mice with the modified allele EM8383, C57BL/6nTac-Zfp365^tm1a(KOMP)Wtsi^/WtsiOrl, were purchased from Wellcome Trust Sanger Institute (Genome Research Limited, Hinxton, Cambridge, UK, CB10 1SA), where briefly the exon 2 is deleted when these mice are crossbred with Cre recombinase positive mice, as this exon is flanked by LoxP sites, thereby generating Tm1b allele [31,32,33,34]. The KO mice for Zfp365 were treated with an intratracheal injection of 40 μL of phosphate-buffered saline (PBS) or bleomycin (Cayman) (4.66 mg/kg of body weight) and sacrificed at 21 days. We used male and female mice between 9–10 weeks of age. Right lungs were fixed with 4% paraformaldehyde for staining with hematoxylin and eosin and Masson’s trichrome stains. Lung sections were coded and scored blindly for the severity and extent of the fibrotic lesions as previously described [35].

### 4.12. Determination of Hydroxyproline

Left lungs from mice were hydrolyzed in 6 N HCl for 24 h at 110 °C. Hydroxyproline was determined by measuring the hydroxyproline content as previously described [36]. Briefly, Aliquots were assayed by adding chloramine T (Sigma-Aldrich, St Louis, MO, USA) solution, followed by development with Erlich reagent. Each sample was tested in duplicate. Data are expressed as μg of hydroxyproline/left lung.

### 4.13. Semiquantitative Evaluation of Lung Morphological Lesions

Lung sections were stained with hematoxylin, eosin, and Masson’s trichrome and scored blindly for severity and extent of the lesions and percentage of fibrosis as previously described [35]. A fibrosis score was determined by multiplying the extent of the lesion × percentage of fibrosis x severity.

### 4.14. Statistical Analyses

All statistical analyses were performed using GraphPad Prism 8.0 Software (La Jolla, CA, USA). All data are expressed as the mean ± standard deviation. Unpaired Student’s *t*-test, one- and two-way ANOVA with Bonferroni correction were performed as indicated for each experiment. A *p*-value < 0.05 was considered statistically significant.

## Data Availability

Data are contained within the article.

## Supplementary Materials

The following supporting information can be downloaded at: https://www.mdpi.com/article/10.3390/cells11182848/s1. Figure S1: *Zfp365* gene expression is absent in K.O. mice; Figure S2: ZNF365 silencing does not affect the apoptotic rate in normal human lung fibroblasts and A549 cells under basal conditions and after an apoptotic stimulus; Figure S3: Silencing ZNF365 promotes the increase in Col1A1 and Col3A1 in human lung fibroblasts; Figure S4: TGFβ-1 stimulation of silenced human lung fibroblasts and A549 cells promotes the expression of senescent markers; Figure S5: Violin plot of the expression of selected genes in epithelial cells obtained from Idiopathic Pulmonary Fibrosis samples (GSE136831 dataset). Ref [37,38,39,40,41,42,43,44] are cited in the supplementary materials.

## References

[1] Selman M., King T.E., Pardo A. (2001). Idiopathic pulmonary fibrosis: Prevailing and evolving hypotheses about its pathogenesis and implications for therapy. Ann. Intern. Med..

[2] King T.E., Pardo A., Selman M. (2011). Idiopathic pulmonary fibrosis. Lancet.

[3] Selman M., Pardo A. (2020). The leading role of epithelial cells in the pathogenesis of idiopathic pulmonary fibrosis. Cell. Signal..

[4] Negreros M., Hagood J.S., Espinoza C.R., Balderas-Martínez Y.I., Selman M., Pardo A. (2019). Transforming growth factor beta 1 induces methylation changes in lung fibroblasts. PLoS ONE.

[5] Renzoni E.A., Abraham D.J., Howat S., Shi-Wen X., Sestini P., Bou-Gharios G., Wells A.U., Veeraraghavan S., Nicholson A.G., Denton C.P. (2004). Gene expression profiling reveals novel TGFβ-1 targets in adult lung fibroblasts. Respir. Res..

[6] Zhang Y., Park E., Kim C.S., Paik J.-H. (2013). ZNF365 promotes stalled replication forks recovery to maintain genome instability. Cell Cycle.

[7] Zhang Y., Shin S.J., Liu D., Ivanova E., Foerster F., Ying H., Zheng H., Xiao Y., Chen Z., Protopopov A. (2013). ZNF365 promotes stability of fragile sites and telomeres. Cancer Discov..

[8] Koyama Y., Hattori T., Shimizu S., Taniguchi M., Yamada K., Takamura H., Kumamoto M., Matsuzaki S., Ito A., Katayama T. (2013). DBZ (DISC1- binding zinc finger protein)-deficient mice display abnormalities in basket cells in the somatosensory cortices. J. Chem. Neuroanat..

[9] Okamoto M., Iguchi T., Hattori T., Matsuzaki S., Koyama Y., Taniguchi M., Komada M., Xie M.-J., Yagi H., Shimizu S. (2015). DBZ regulates cortical cell positioning and neurite development by sustaining the anterograde transport of Lis1 and DISC1 through control of Ndel1 dual phosphorylation. J. Neurosci..

[10] Hirohashi Y., Wang Q., Liu Q., Li B., Du X., Zhang H., Furuuchi K., Masuda K., Sato N., Greene M.I. (2006). Centrosomal proteins Nde1 and Su48 form a complex regulated by phosphorylation. Oncogene.

[11] Bolaños A.L., Milla C.M., Lira J.C., Ramírez R., Checa M., Barrera L., García-Álvarez J., Carbajal V., Becerril C., Gaxiola M. (2012). Role of Sonic hedgehog in idiopathic pulmonary fibrosis. Am. J. Physiol. Lung Cell. Mol. Physiol.

[12] Sanders Y.Y., Ambalavanan N., Halloran B., Zhang X., Liu X., Crossman D.K., Bray M., Zhang K., Thannickal V.J., Hagood J.S. (2012). Altered DNA methylation profile in idiopathic pulmonary fibrosis. Am. J. Respir. Crit. Care Med..

[13] Casella G., Munk R., Kim K.M., Piao Y., De S., Abdelmohsen K., Gorospe M. (2019). Transcriptome signature of cellular senescence. Nucleic Acids Res..

[14] Horowitz J.C., Rogers D.S., Sharma V., Vittal R., White E.S., Cui Z., Thannickal V.J. (2007). Combinatorial activation of FAK and AKT by transforming growth factor-β-1 confers and anoikis resistant phenotype to myofibroblasts. Cell. Signal..

[15] Lee J.S., La J., Aziz S., Dobrinskikh E., Brownell R., Jones K.D., Achtar-Zadeh N., Green G., Elicker B.M., Golden J.A. (2021). Molecular markers of telomere dysfunction and senescence are common findings in the usual interstitial pneumonia pattern of lung fibrosis. Histopathology.

[16] Álvarez D., Cárdenes N., Sellarés J., Bueno M., Corey C., Hanumanthu V.S., Peng Y., D’Cunha H., Sembrat J., Nouraie M. (2017). IPF lung fibroblasts have a senescent phenotype. Am. J. Physiol. Lung Cell. Mol. Physiol..

[17] Reyfman P.A., Walter J.M., Joshi N., Anekalla K.R., McQuattie-Pimentel A.C., Chiu S., Fernandez R., Akbarpour M., Chen C.-I., Ren Z. (2019). Single-Cell transcriptomic analysis of human lung reveals complex multicellular changes during pulmonary fibrosis. Am. J. Respir. Crit. Care Med..

[18] Morse C., Tabib T., Sembrat J., Buschur K., Trejo Bittar H., Valenzi E., Jiang Y., Kass D.J., Gibson K., Chen W. (2019). Proliferating SPP1/MERTK-expressing macrophages in idiopathic pulmonary fibrosis. Eur. Respir. J..

[19] Adams T.S., Schupp J.C., Poli S., Ayaub E.A., Neumark N., Ahangari F., Chu S.G., Raby B.A., DeIuliis G., Janusyk M. (2019). Single Cell RNA-seq reveals extopic and aberrant lung resident cell populations in idiopathic pulmonary fibrosis. Sci. Adv..

[20] Habermann A.C., Gutiérrez A.J., Bui L.T., Yahn S.L., Winters N.I., Calvi C.L., Peter L., Chung M.-I., Taylor C.J., Jetter C. (2020). Single-Cell RNA sequencing reveals profibrotic roles of distinct epithelial and mesenchymal lineages in pulmonary fibrosis. Sci. Adv..

[21] Yu G., Kovkarova-Naumovski E., Jara P., Parwani A., Kass D., Ruiz V., López-Otín C., Rosas I.O., Gibson K.F., Cabrera S. (2012). Matrix metalloproteinase-19 is a key regulator of lung fibrosis in mice and humans. Am. J. Respir. Crit. Care Med..

[22] Parra E.R., Lin F., Martins V., Rangel M.P., Capelozzi V.L. (2013). Immunohistochemical and morphometric evaluation of COX-1 and COX-2 in the remodeled lung in idiopathic pulmonary fibrosis and systemic sclerosis. J. Bras. Pneumol..

[23] Keerthisingam C.B., Jenkins R.G., Harrison N.K., Hernandez-Rodriguez N.A., Booth H., Laurent G.J., Hart S.L., Foster M.L., McAnulty R.J. (2001). Cyclooxygenase-2 deficiency results in a loss of the anti-proliferative response to transforming growth factor-beta in human fibrotic lung fibroblasts and promotes bleomycin-induced pulmonary fibrosis in mice. Am. J. Pathol..

[24] González-Gualda E., Baker A.G., Fruk L., Muñoz-Espín D. (2020). A guide to assess cellular senescence in vitro and in vivo. FEBS J..

[25] Di Micco R., Krizhanovsky V., Baker D., di Fagagna F.D. (2021). Cellular senescence in ageing: From mechanisms to therapeutic opportunities. Nat. Rev. Mol. Cell Biol..

[26] Pardo A., Selman M. (2020). The interplay of the genetic architecture, aging and environmental factors in the pathogenesis of idiopathic pulmonary fibrosis. Am. J. Respir. Cell Mol. Biol..

[27] Yao C., Guan X., Carraro G., Parimon T., Liu X., Huang G., Mulay A., Soukiasian H.J., David G., Weigt S.S. (2021). Senescence of alveolar type 2 cells drives progressive pulmonary fibrosis. Am. J. Respir. Crit. Care. Med..

[28] Arcamone F., Animati F., Cuprunico G., Lombardi P. (1997). New developments in antitumor antracyclines. Pharmacol. Ther..

[29] Bojko A., Czarnecka-Herok J., Charzynska A., Dabrowski M., Sikora E. (2019). Diversity of the senescence phenotype of cancer cells treated with chemotherapeutic agents. Cells.

[30] Da Silveira W.A., Renaud L., Hazard E.S., Hardiman G. (2022). MiRNA and lncRNA expression networks modulate cell cycle and DNA repair inhibition in senescent prostate cells. Genes.

[31] White J.K., Gerdin A.-K., Karp N.A., Ryder E., Buljan M., Bussell J.N., Salisbury J., Clare S., Ingham N.J., Podrini C. (2013). Genome-wide generation and systematic phenotyping of knockout mice reveals new roles for many genes. Cell.

[32] Skarnes W.C., Rosen B., West A.P., Koutsourakis M., Bushell W., Iyer V., Mujica A.O., Thomas M., Harrow J., Cox T. (2011). A conditional knockout resource for the genome-wide study of mouse gene function. Nature.

[33] Bradley A., Anastassiadis K., Ayadi A., Battey J.F., Bell C., Birling M.-C., Bottomley J., Brown S.D., Bürger A., Bult C.J. (2012). The mammalian gene function resource: The international knockout mouse consortium. Mamm. Genome.

[34] Pettitt S.J., Liang Q., Rairdan X.Y., Moran J.L., Prosser H.M., Beier D.R., Lloyd K.C., Bradley A., Skarnes W.C. (2009). Agouti C57BL/6N embryonic stem cells for mouse genetic resources. Nat. Methods.

[35] Pardo A., Barrios R., Gaxiola M., Segura-Valdez L., Carrillo G., Estrada A., Mejía M., Selman M. (2000). Increase of lung neutrophils and upregulation of neutrophil gelatinase B and collagenase in hypersensitivity pneumonitis. Am. J. Respir. Crit. Care Med..

[36] Cabrera S., Maciel M., Herrera I., Nava T., Vergara F., Gaxiola M., López Otín C., Selman M., Pardo A. (2015). Essential role for the ATG4B protease and autophagy in bleomycin-induced pulmonary fibrosis. Autophagy.

[37] Adams, T.S., Schupp J.C., Poli S., Ayaub E.A. (2020). Single-cell RNA-seq reveals ectopic and aberrant lung-resident cell populations in idiopathic pulmonary fibrosis. Sci. Adv..

[38] Team R.C. (2013). Others. R: A language and environment for statistical computing. Sci. Adv..

[39] Gentleman R.C., Carey V.J., Bates D.M., Bolstad B., Dettling M., Dudoit S. (2004). Bioconductor: Open software development for computational biology and bioinformatics. Genome Biol..

[40] Hao Y. (2021). Integrated analysis of multimodal single-cell data. Cell.

[41] Wickham Y. (2016). ggplot2: Elegant Graphics for Data Analysis.

[42] https://www.infrafrontier.eu/sites/infrafrontier.eu/files/upload/public/pdf/Resources%20and%20Services/eucomm-alleles-overview_infrafrontier-2016.pdf.

[43] https://www.infrafrontier.eu/sites/infrafrontier.eu/files/upload/public/pdf/Resources%20and%20Services/eucomm_komp-csd_allele_conversion_guide_v3a_2016.pdf.

[44] https://www.infrafrontier.eu/sites/infrafrontier.eu/files/upload/public/pdf/genotype_protocols/EM08383_geno.pdf.

