# Supplementary material for: Lack of ZNF365 Drives Senescence and Exacerbates Experimental Lung Fibrosis
Source: Cells. 2022 Sep 13;11(18):2848. doi: 10.3390/cells11182848 (PMC9497065; doi:10.3390/cells11182848)
Supplement: Supplementary file 1 [file cells-11-02848-s001.zip › cells-1852886-supplementary.pdf]

Supplementary Information

Lack of ZNF365 Drives Senescence and Exacerbates Experimental Lung Fibrosis

Juan Urista[1], Mariel Maldonado[2], Fernanda Toscano-Marquez[1], Remedios Ramírez[1], Yalbi Itzel Balderas-Martínez[2] Carina Becerril[2], Yair Romero[1], Moisés Selman[2], Annie Pardo[1].

1 Facultad de Ciencias, Universidad Nacional Autónoma de México, México, Mexico.

2 Instituto Nacional de Enfermedades Respiratorias, "Ismael Cosío Villegas", Mexico, Mexico.

Correspondence should be addressed to: apardos@unam.mx

SUPPLEMENTARY METHODS

Single-cell RNA expression analysis

Single-cell expression data were downloaded from GEO accession number GSE136831 [1] that had been previously annotated. We used R software version 4.1.0 [2], with Bioconductor version 3.13 [3]. We performed the normalization, scaling, Principal Component Analysis (PCA), and Uniform Manifold Approximation and Projection (UMAP) using the Seurat package version 4.1.1 [4]. Graphics were done using ggplot2 version 3.3.6 [5].

Real-time polymerase chain reaction (PCR)

Supplementary reactions were performed using specific Taqman probes (Applied Biosystems) for Col1a1 (Hs00164004_m1), Col3a1 (Hs00943809_m1), and POLR2A (Hs00172187_m1), or specific primer sequences and SybrGreen reagent

(ThermoFisher Scientific, catalog #K0222). QuantStudio 6 (ThermoFisher Scientific) thermalcycler was used for qPCR experiments, and 2-ΔCt was used for data analysis. We provide primer sequences in the table below.

**Name**	**Sequence (5' -> 3')**	**oligo length (nt)**
Hu CDKN1A-F	TGTCCGTCAGAACCCATGC	19
Hu CDKN1A-R	AAAGTCGAAGTTCCATCGCTC	21
Hu HPRT-R1	GGCTTTGTATTTTGCTTTTCCA	22
Hu HPRT-F1	AAGGACCCCACGAAGTGTTG	20
Hu TP53-F	CAGCACATGACGGAGGTTGT	20
Hu TP53-R	TCATCCAAATACTCCACACGC	21

Genotyping

Endpoint PCRs were performed according to the transgenic mice providers. Specific primers given in the technical sheet were designed and synthesized to detect the WT and KO mice strains [6-8].

SUPPLEMENTARY FIGURES

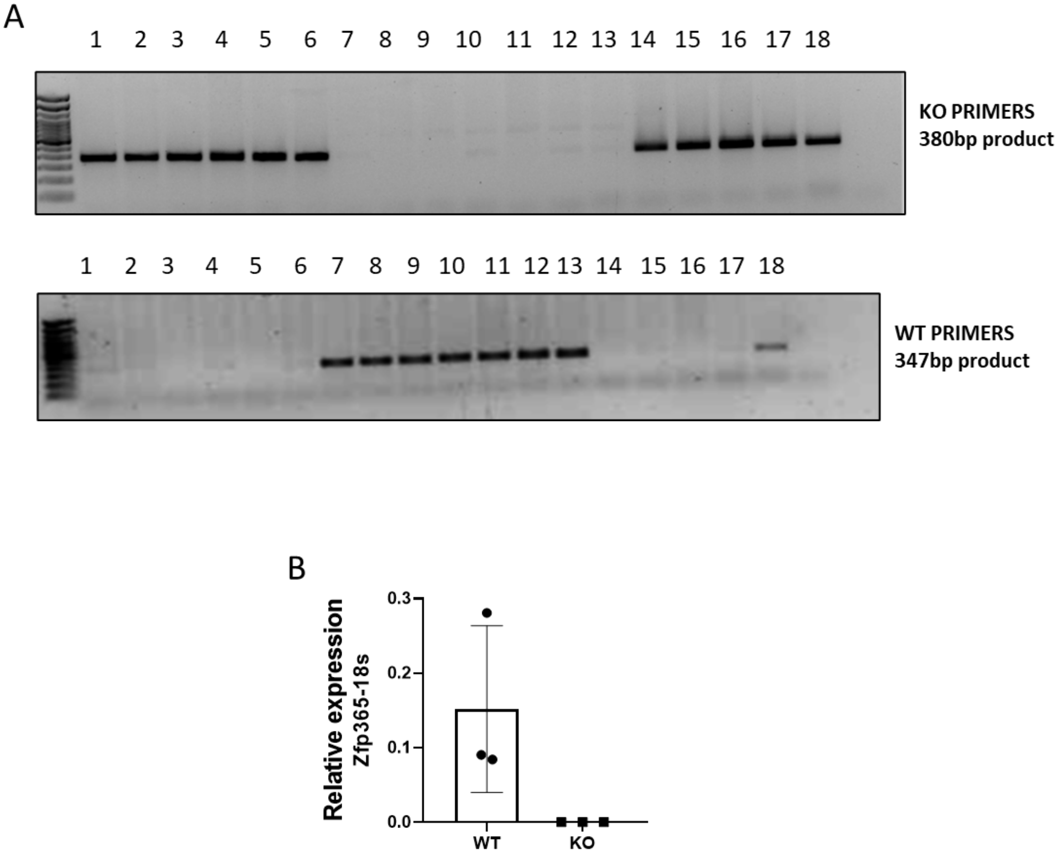

Supplementary Figure S1. A. DNA electrophoresis gel images with an example of the genotyping of WT (7-13) and KO (1-6; 14-17 and 18 is HT) mice. Each lane is numbered and represents one animal. WT oligonucleotides amplify a segment of the ZNF365 gene, while KO oligonucleotides amplify the cassette that disrupts *ZNF365* gene. B. PCR of relative expression of *ZNF365*.

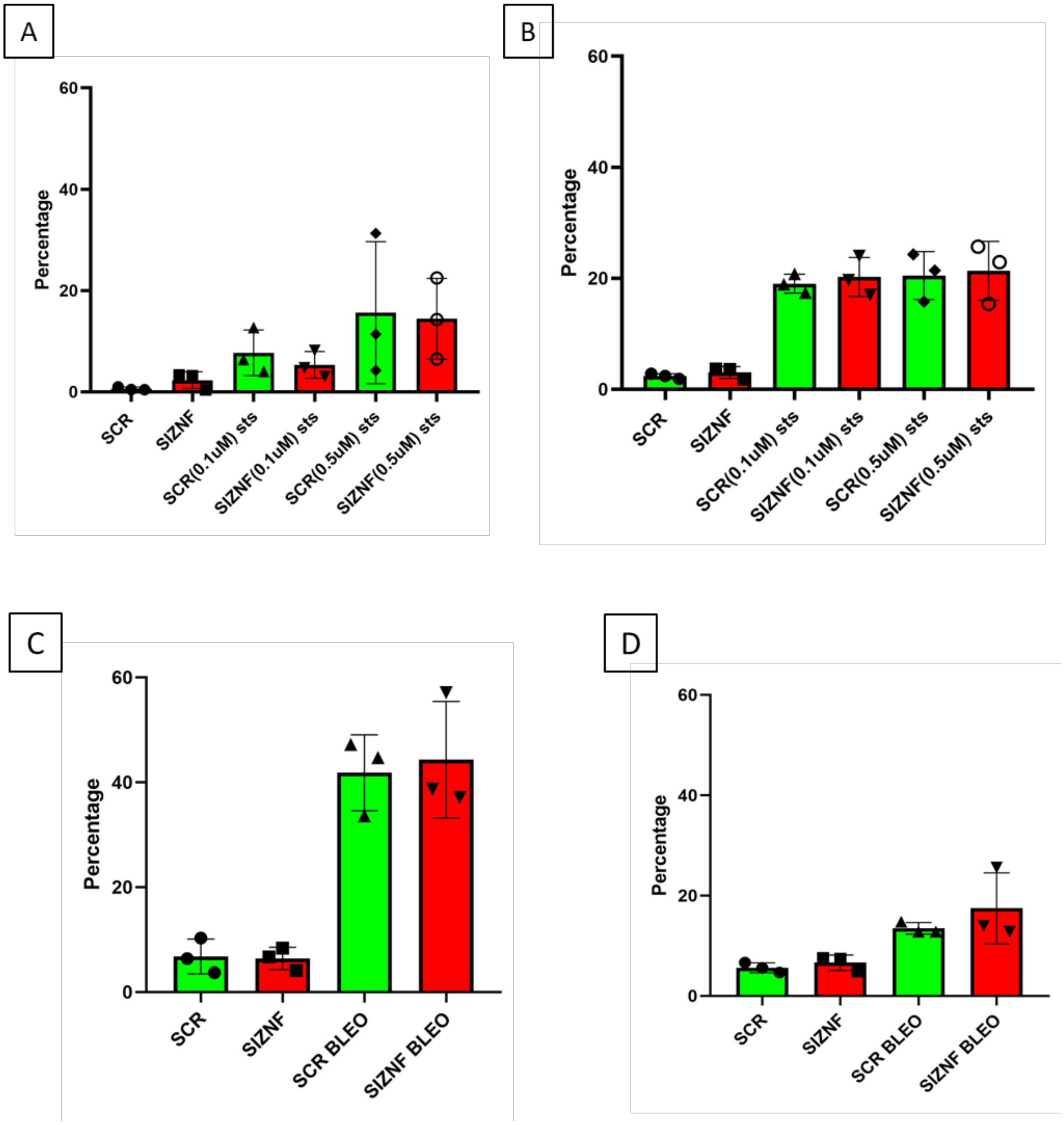

Supplementary Figure S2. ZNF365 silencing does not affect the apoptotic rate in normal human lung fibroblasts and A549 cells under basal conditions and after an apoptotic stimulus. A and B: Early and late percentage of basal apoptosis in normal human lung fibroblasts under basal conditions and after 0.1 μM and 0.5 μM staurosporine stimulation. C and D: Early and late percentage of apoptosis in A549 epithelial cells under basal conditions and after 30mU of bleomycin stimulation.

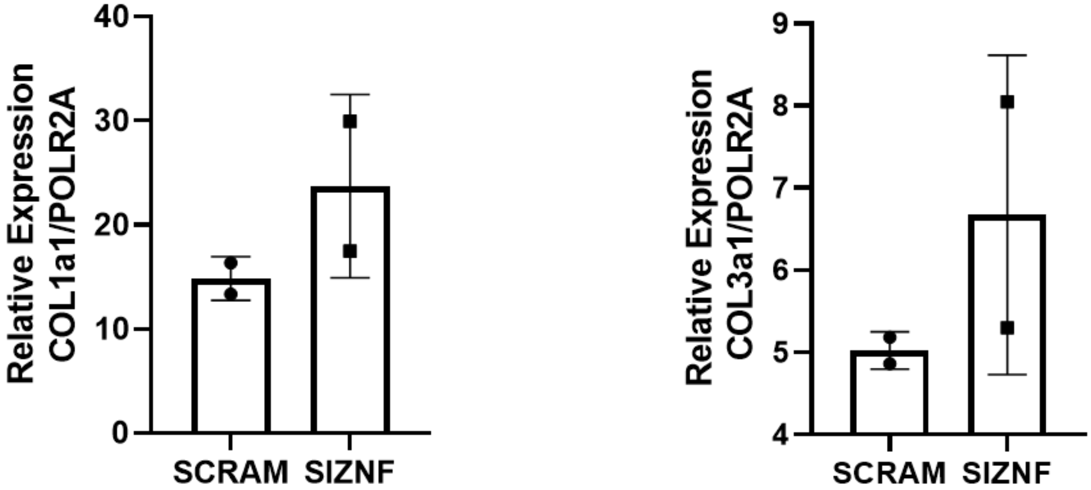

Supplementary Figure S3. Silencing ZNF365 promotes the increase of *Col 1A1* and *Col 1A3* in human lung fibroblasts. Figures represent the mean +/- S.D. of two independent experiments.

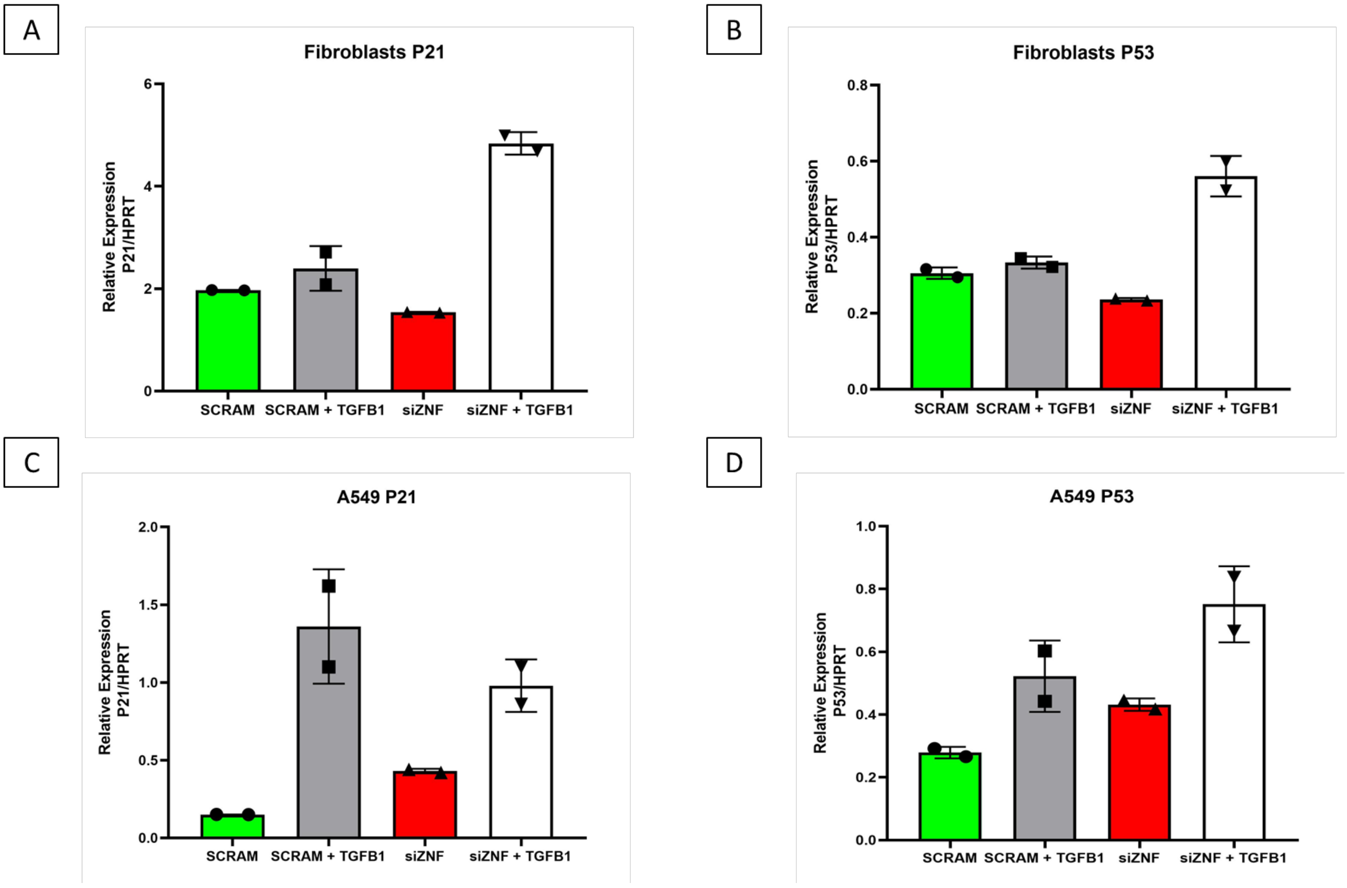

Supplementary Figure S4. TGFβ-1 stimulation of silenced A549 cells and human lung fibroblasts promotes the expression of senescent markers. A and B) *p21* and *p53* expression in normal human lung fibroblasts. C and D) *p21* and *p53* expression in A549 cells. Figures represent the mean +/- S.D. of two independent experiments.

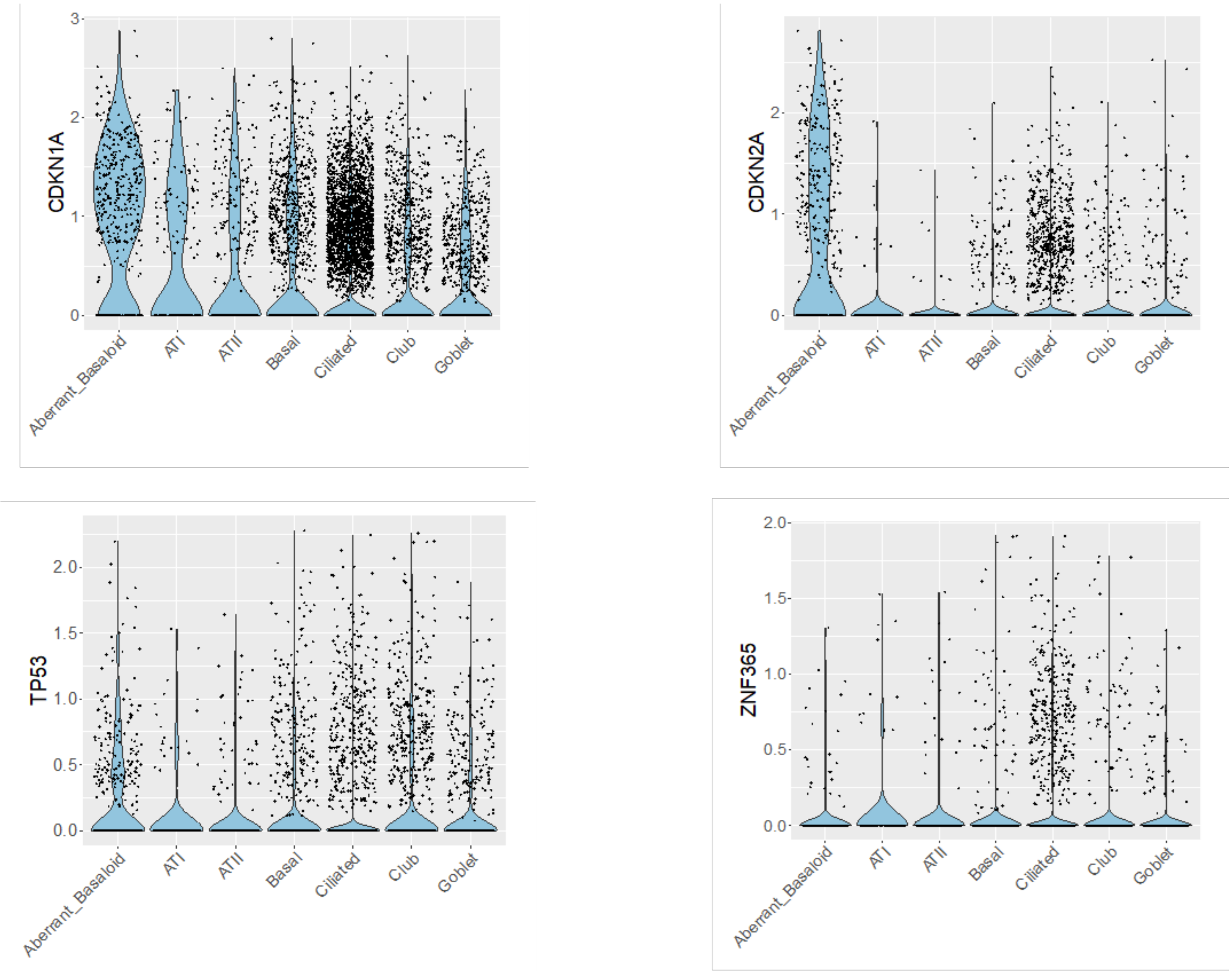

Supplementary Figure S5. Violin plot of the expression of selected genes in epithelial cells obtained from Idiopathic Pulmonary Fibrosis samples (GSE136831 dataset).

References

1.Adams, T.S.; Schupp, J.C.; Poli, S.; Ayaub, E.A. Single-cell RNA-seq reveals ectopic and aberrant lung-resident cell populations in idiopathic pulmonary fibrosis. *Sci. Adv.* **2020**, *6*, eaba1983.

2. Team, R.C. Others. R: A language and environment for statistical computing. 2013

3.Gentleman, R.C.; Carey, V.J.; Bates, D.M.; Bolstad, B.; Dettling, M.; Dudoit, S. Bioconductor: open software development for computational biology and bioinformatics. *Genome Biol.* **2004**, *5*, R80.

4. Hao, Y. Integrated analysis of multimodal single-cell data. *Cell* **2021**, 184, 3573 - 3587.e29

5.Wickham, H. *ggplot2: Elegant Graphics for Data Analysis*; Springer-Verlag New York, NY, USA, 2016.

6.**https://www.infrafrontier.eu/sites/infrafrontier.eu/files/upload/public/pdf/Resources%20and%20Services/eucomm-alleles-overview_infrafrontier-2016.pdf (accessed on 20 July 2022)**

7.**https://www.infrafrontier.eu/sites/infrafrontier.eu/files/upload/public/pdf/Resources%20and%20Services/eucomm_komp-csd_allele_conversion_guide_v3a_2016.pdf (accessed on 20 July 2022)**

8.**https://www.infrafrontier.eu/sites/infrafrontier.eu/files/upload/public/pdf/genotype_protocols/EM08383_geno.pdf (accessed on 20 July 2022)**
